# Potential Implications of Natural Antioxidants of Plant Origin on Oxidative Stability of Chicken Albumen during Storage: A Review

**DOI:** 10.3390/antiox11040630

**Published:** 2022-03-25

**Authors:** Uchechukwu Edna Obianwuna, Vivian U. Oleforuh-Okoleh, Jing Wang, Hai-Jun Zhang, Guang-Hai Qi, Kai Qiu, Shu-Geng Wu

**Affiliations:** 1 National Engineering Research Center of Biological Feed, Institute of Feed Research, Chinese Academy of Agricultural Sciences, Beijing 100081, China; 2019y90100102@caas.cn (U.E.O.); wangjing@caas.cn (J.W.); zhanghaijun@caas.cn (H.-J.Z.); qiguanghai@caas.cn (G.-H.Q.); 2 Department of Animal Science, Faculty of Agriculture, Rivers State University, Nkpolu-Oroworukwo, Port-Harcourt PMB-5080, Nigeria; vivian.oleforuh-okoleh@ust.edu.ng

**Keywords:** egg, albumen quality, storage time and temperature, plants, natural antioxidants

## Abstract

Enhanced albumen quality is reflected in increased thick albumen height, albumen weight, and Haugh unit value, while the antimicrobial, antioxidant, foaming, gelling, viscosity, and elasticity attributes are retained. Improved albumen quality is of benefit to consumers and to the food and health industries. Egg quality often declines during storage because eggs are highly perishable products and are most often not consumed immediately after oviposition. This review provides insights into albumen quality in terms of changes in albumen structure during storage, the influence of storage time and temperature, and the mitigation effects of natural dietary antioxidants of plant origin. During storage, albumen undergoes various physiochemical changes: loss of moisture and gaseous products through the shell pores and breakdown of carbonic acid, which induces albumen pH increases. High albumen pH acts as a catalyst for structural changes in albumen, including degradation of the β-ovomucin subunit and *O*-glycosidic bonds, collapse of the ovomucin-lysozyme complex, and decline in albumen protein–protein interactions. These culminate in declined albumen quality, characterized by the loss of albumen proteins, such as ovomucin, destabilized foaming and gelling capacity, decreased antimicrobial activity, albumen liquefaction, and reduced viscosity and elasticity. These changes and rates of albumen decline are more conspicuous at ambient temperature compared to low temperatures. Thus, albumen of poor quality due to the loss of functional and biological properties cannot be harnessed as a functional food, as an ingredient in food processing industries, and for its active compounds for drug creation in the health industry. The use of refrigerators, coatings, and thermal and non-thermal treatments to preserve albumen quality during storage are limited by huge financial costs, the skilled operations required, environmental pollution, and residue and toxicity effects. Nutritional interventions, including supplementation with natural antioxidants of plant origin in the diets of laying hens, have a promising potential as natural shelf-life extenders. Since they are safe, without residue effects, the bioactive compounds could be transferred to the egg. Natural antioxidants of plant origin have been found to increase albumen radical scavenging activity, increase the total antioxidant capacity of albumen, reduce the protein carbonyl and malondialdehyde (MDA) content of albumen, and prevent oxidative damage to the magnum, thereby eliminating the transfer of toxins to the egg. These products are targeted towards attenuating oxidative species and inhibiting or slowing down the rates of lipid and protein peroxidation, thereby enhancing egg quality and extending the shelf life of albumen.

## 1. Introduction

Eggs are a common source of animal protein because they are inexpensive compared with other sources and contain bioactive substances with high biological value [1]. The various components, such as shell, albumen, and yolk, control egg quality. Albumen contains proteins (mainly ovalbumin, ovotransferrin, ovomucoid, lysozyme, and ovomucin), peptides, and amino acids (AA), which are natural antioxidant compounds [2]. Albumen possesses biological and functional properties, owing to its structure and protein content. The functional properties of albumen, such as foaming, emulsifying and gelling, are significant in the food industry [3]; for instance, foaming stability is beneficial for enhancing the quality of aerated food products [4]. Proteins, more often in powdered form, are used in the food industry. For example, albumen is used mainly in the form of powdered protein in the production of marshmallows, hot ice cream, meringues, creams, and bakery products, while the yolk is used for the production of dressings, mayonnaises, and sauces [5]. Albumen peptides are also considered functional foods and are used as ingredients in human drugs due to their biological functions [6]. However, eggs are highly perishable products and their quality is lost when they are not properly handled and stored. Storage time and temperature are critical factors that affect the deterioration of albumen quality [7].

During storage, eggs undergo some physiochemical changes, such as loss of moisture and gases (mainly oxygen and carbon dioxide) through the eggshell pores and egg membranes, loss of moisture content, and increases in albumen pH, as well as increases in the size of air cells [8] and flattening of the yolk due to movement of water from the albumen through the weaker vitelline membrane into the yolk [9]. Additionally, the fraction of the thick gelatinous albumen becomes reduced and less jelly-like, which may be due to changes in the ovomucin–lysozyme complex [10,11]. Among the biochemical changes in eggs during storage, protein oxidation occurs within the albumen, altering physicochemical and functional properties [12]. All of these factors culminate in the loss of functional properties of albumen and, consequently, low albumen quality.

Accordingly, parameters such as albumen pH, thick albumen height, thick albumen index, egg weight, and Haugh unit value (HU), which is calculated based on thick albumen height and egg weight, have been used as indicators for measuring albumen quality based on egg freshness [13]. Extended storage time causes a reduction in thick albumen content, which is reflected in lower HU values [14,15], whereas albumen pH increases owing to a shift in the equilibrium of the bicarbonate buffer system [16]. It has been shown that the decline in albumen quality during storage is more rapid at higher temperatures compared to lower temperatures [17]. The preservation of albumen quality is critical for producers, consumers, and the food industry. Several studies have addressed this issue in the literature.

A number of approaches have been adopted to mitigate the adverse effects of storage on albumen quality. Such strategies include washing the eggshell to reduce the microbial load on the shell surface, though this damages the cuticle layer [11], and the use of pulsed light [18] and ultraviolet light radiation [19] to decontaminate the shell surface, though the functional properties of the albumen may be altered. In addition, ultrasound treatment [4], modified atmosphere packaging [20], and the use of slightly acidic electrolysed water [21] have been found to preserve albumen quality during storage. However, the use of these methods is limited by the skilled operations they involve and substantial financial costs. Coatings with natural products, such as shellac [22], glycerine oil [23,24], citric acid [24], and whey protein [25], extend the shelf life of eggs without the risk of toxicity, dosage use, or environmental pollution. Hence, natural antioxidants of plant origin may be promising alternatives to synthetic products for preserving albumen quality during storage and improving consumer acceptance.

Natural antioxidants that are plant-derived products are not only characterized by being multifunctional and less toxic, they have also been found to improve egg quality and the antioxidant capacity of laying hens [26] when incorporated into animal feed. Hence, they are considered safe in terms of their chemical structure. Natural antioxidants of plant origin contain bioactive compounds (phenols, carotenoids, anthocyanins) [27] which can be transferred to eggs. Therefore, the critical value of the application of natural antioxidants lies in their capacity to suppress oxidation processes and reduce the levels of oxidative products in the animal and food system [28]. Previous studies have shown that natural dietary antioxidants supplemented in the diets of laying hens can preserve the shelf life of albumen during storage [29,30]. Natural antioxidants of plant origin, including acai lump flour [31], natural astaxanthin [32], pumpkin seeds [30], green tea [29], and rapeseed oil [33], preserved the shelf life of albumen during storage.

Much attention has been given to the application of plants (leaves, seed, and oil) as feed additives to improve performance and, to some extent, the quality of freshly laid eggs. However, limited data are available on the effects of phyto additives incorporated into the diets of laying hens and on the preservation of egg quality during storage. Therefore, this review provides insights into albumen quality in terms of changes in albumen structure during storage, the influence of storage time and temperature, and the mitigation effects of natural dietary antioxidants of plant origin.

## 2. Structural Changes in Albumen Quality during the Storage Period

The egg storage environment influences the nutritional and functional properties of albumen. Changes in albumen quality during storage are characterized by various physical and chemical reactions that are affected by either an increase or a decrease in storage time and temperature. Changes in albumen structure indicate a decline in albumen quality during storage. Albumen traits, such as HU value, albumen pH, and thick albumen height, are often used as indicators of albumen quality during storage and are influenced by storage conditions in a time- and temperature-dependent manner [17,34,35]. These traits would invariably influence the albumen’s functional, rheological, and biological properties [10,36]. We reviewed the effects of storage time and temperature on albumen quality and, consequently, the functional properties of the albumen.

### 2.1. Egg Weight Loss

During storage, egg weight loss occurs and influences the egg components (albumen and yolk). The rate of egg weight loss is a critical indicator for evaluating the freshness of eggs [37] and is associated with the economic value of eggs. Egg weight loss is attributed to the loss of moisture and carbon dioxide through shell pores [9]. The rate of gaseous and moisture escape from the shell pores during storage depends on the storage environment (temperature 4 °C or >27 °C, relative humidity, and air flow). Previous studies [34,38] revealed that egg weight loss is influenced by storage time and temperature. Extended storage time at ambient temperature increases the loss of moisture and gases from the egg to the environment [17]. It has been reported that egg weight loss is higher under ambient temperature compared to refrigerated temperatures [7,34,38]. The lower egg weight loss observed at refrigerated temperatures could be linked to the fast drying and shrinkage of the cuticle plugging air pores in the eggshell. In addition, there is less loss of solvents (moisture and gaseous products) from egg contents, in contrast to storage at ambient temperature, at which the size of air pores increases, facilitating the escape of moisture and carbon dioxide [39]. However, Samli et al. [8] reported that cold storage did not affect egg weight loss. Reduced rates of egg weight loss during storage helps maintain the internal quality of eggs during extended storage periods. The escape of gases and moisture through shell pores, leading to egg weight loss, also causes changes in albumen pH.

### 2.2. Albumen pH

Albumen pH (ApH) is a helpful indicator for evaluating changes in albumen quality over storage time. ApH is about 7.6 in fresh eggs, and the optimum pH of albumen ranges from 7.5–8.50 [3]; however, it increases during the storage period and reaches 9.5. Previous studies have shown that ApH increases with extended storage time [14,40] and storage temperatures [3,22]. Albumen pH increases more at room temperature than at low temperatures [41,42]. Škrbić et al. [43] reported that refrigerated temperatures lower the rate of increase in albumen pH compared to ambient temperature. However, Altunatmaz et al. [35] found no significant effect of temperature on albumen pH after 28 d of storage. The increased pH at room temperature could be due to the increased escape of gases from eggs. Studies that have reviewed the effects of storage time and temperature on ApH are presented in Table 1.

The studies presented in Table 1 revealed that, during storage, ApH increases above the ApH of fresh eggs (9.8 vs. 8.0%) owing to time and temperature effects. This increase could be due to the dissociation of carbonic acid (H_2_CO_3_) leading to the formation of water and CO_2_ within the albumen and the escape of CO_2_ into the environment via the shell pores. This leads to the alkalization of the albumen and a shift in the bicarbonate buffer equilibrium [50]. The increased alkalinity of albumen causes a decrease in ovomucin content [12], and the highly viscous thick albumen adjacent to the yolk progressively loses its gelatinous structure, leading to albumen liquefaction [51]. Albumen pH influences the strength of the vitelline membrane, and increased pH values weaken the vitelline membrane, which may facilitate a high loss of gas and moisture from eggshell pores. Feddern et al. [17] reported that increased ApH negatively affects the vitelline membrane and hastens the exchange of albumen alkaline ions with yolk H^+^, leading to protein denaturation. All of these factors cause a decline in albumen quality, and the decline is more conspicuous at ambient temperature than at low temperatures, irrespective of time; hence, ApH is a function of storage time and temperature. Albumen thinning due to increased pH levels is reflected in albumen height.

### 2.3. Albumen Height

Albumen height (AH) is fundamental for calculating the albumen index and Haugh unit values used for albumen quality evaluation. Albumen height is at a maximum in freshly laid eggs and declines with storage time. Various studies [40,52] have demonstrated that AH decreases with storage time and, in one study, AH and albumen index could not be calculated after 18 days of storage owing to increased albumen fluidity [35]. The effects of storage time and temperature on albumen height are presented in Table 2.

In all 20 studies examined, there was a 100% indication that albumen height decreased significantly during storage relative to the albumen height of fresh eggs. The reduction in albumen height during storage was due to liquefaction of the thick albumen. A decrease in thick albumen height was evident in eggs stored at room temperature compared with those stored at low temperatures [14,40,42]. Albumen proteins, which are fundamental to AH, are decreased quantitatively during storage due to temperature effects [12,56], thereby contributing to the decreased AH observed during storage. The other albumen indices were also affected by storage. Extended storage time decreased albumen weight [14,57] and albumen percentage [58]. This decrease in albumen weight was due to a decrease in the thick albumen weight and an increase in yolk weight, suggesting that water diffused from the albumen through the vitelline membrane into the yolk during storage. Albumen weight loss is higher at room temperature than under low temperatures [35] because of higher water loss from the albumen to the yolk. A drastic reduction in AH over storage time indicates poor albumen quality and adversely influences HU values.

### 2.4. Haugh Unit Value

It has been established that albumen quality and egg freshness can be measured based on Haugh unit (HU) values, and HU is determined from thick albumen height and egg weight. The effects of storage time and temperature on HU values are listed in Table 3.

Various studies [17,32,42,53] have reported lower HU values in stored eggs than in fresh eggs. The following are reported comparisons of the HU values of fresh and stored eggs: 82.17 vs. 0 for eggs stored at 21 °C for 30 days [42], 79.21 vs. 62.82 for eggs stored for at 30 °C for 15 days [53], 98.6 vs. 39.36 for eggs stored at 33 °C for 63 days [17], and 83.5 vs. 0 for eggs stored at 25 °C for 56 days [32]. The decline in HU values of stored eggs is due to the disintegration of the ovomucin–lysozyme complex, proteolysis of dense proteins, and consequent reduction in the height of thick albumen [61]. The decrease in HU values was more rapid in eggs stored at ambient temperature than in eggs stored at refrigerated temperatures. The following are reported HU values of eggs at room and refrigerated temperatures when stored for 28 days: 35 vs. 74 [56], 32.66 vs. 71.6 [14], 32.71 vs. 56.41 [46] 38 vs. 64.77 [43], and 0 vs. 74.48 [35]. Although no decrease was observed at a temperature of 5 °C [14], this is in agreement with the reports of [17,46].

The quality of stored eggs is graded according to HU score value: AA grade > 72, A grade = 60–72, B grade < 60, and C grade < 30 [62]. The HU grade of eggs stored at refrigerated temperatures was found to be higher than that of eggs stored at ambient temperature: AA vs. 0 [35], A vs. C [43], and AA vs. 0 [32]. However, Souza et al. [41] reported that, after storage, eggs stored at both temperatures were classified as AA grade, although the HU value of eggs stored at refrigerated temperatures were numerically higher compared to those stored at ambient temperature. The higher HU value and grade of eggs stored at refrigerated temperatures indicates better egg quality, probably because albumen quality is preserved to a greater extent. The lower HU value of eggs stored at ambient temperature could be linked to faster degradation of the ovomucin–lysozyme complex and rapid liquefaction of dense albumen [38]. Hence, albumen thinning occurs more at room temperature than at low temperatures [11]. HU was found to be more stable with less variation under refrigerated conditions than at ambient temperature, suggesting that storage temperature may be a more critical factor influencing HU.

Decreased HU values during storage suggests loss of albumen functional properties. Albumen consistency was lost when the HU score was <70 during storage [25]. Eggs with lower HU values showed a significant reduction in the viscosity and elasticity modulus of ovomucin gel and disaggregation of *O*-glycoside bonds in ovomucin [63,64]. Therefore, it is crucial to maintain a high HU value during storage. HU values above 70 indicate low protein and lipid peroxidation and, hence, better albumen quality during storage and vice versa.

### 2.5. The Effects of Storage Time and Temperature on Functional Properties of the Albumen

The effects of storage time and temperature on albumen traits (ApH, AH, and HU) alter albumen structure and, consequently, influence the biological and functional properties of the albumen.

Albumen viscosity determines its functional properties, such as emulsification, whippability, and gelling properties [65]. Owing to storage time and temperature effects, changes in albumen structure may influence albumen viscosity; consequently, these functional properties are lost when albumen quality declines. The studies of [10,29] demonstrated that albumen viscosity decreases with storage time. Wang et al. [10] reported that, during an extended storage time, beyond a certain point, no changes were observed in viscosity measurement, showing that the gelatinous nature of the albumen had completely disappeared. In another study, eggs were stored at 24 °C for six weeks and the albumen viscosities of fresh and stored eggs was reported as 75.11 vs. 7.72 [22] and 60.46 vs. 5.73 [3]. Reduced albumen viscosity during storage may be attributed to the destabilization of the *O*-glycoside link between trisaccharides, collapse of the ovomucin gel structure [65], flow behavior index of the albumen over temperature ranges [66], and release of bound water molecules due to the hydrolysis of amino acid chains by the enzymes in the albumen. Reduced albumen viscosity corresponds to an increase in albumen fluidity, reflecting increased total soluble solids [67] and increased liquefaction of the yolk, which subsequently diffuses into the albumen. In addition, decreased viscosity due to storage time reduces the foaming properties of the albumen [3]. When albumen viscosity is lost, the thick albumen becomes less dense and causes albumen liquefaction (albumen thinning).

To a great extent, albumen thinning indicates a decline in egg quality. The reduction in the viscosity of thick albumen gel leads to deterioration in the gelling property and, consequently, albumen thinning [12,68]. Omana et al. [61] demonstrated that albumen thinning was evident at a later time of storage compared to the initial time, suggesting that there is a progressive loss of thick albumen gel with increasing storage time; hence, albumen thinning may be a function of storage time. In addition, albumen thinning has also been associated with the sulfhydryl (SH) groups of ovalbumin, which undergo transition and conversion during storage [69]. S-ovalbumin, a conformational isomer of native ovalbumin, was irreversibly transformed from ovalbumin during prolonged egg storage [13]. This change was due to the configurational inversion of amino acid residues, which is critical for the formation of thermo-stabilized ovalbumin [70]. A higher content of S-ovalbumin increases albumen liquefaction—a claim that is supported by the study of Huang et al. [13], which reported a negative correlation between HU value and S-ovalbumin content. This is in accordance with the study of Fu et al. [71], which revealed that a higher content of S-ovalbumin indicates a lower HU value and reduced freshness of eggs. In addition, albumen thinning may be due to reduced ovomucin–lysozyme interactions and degradation of ovalbumin or clusterin due to proteolysis associated with increased pH [61]. Yuceer and Caner [9] explained that protease enzymes, depolymerized by hydroxyl ions at increasing pHs, destabilize the ovomucin–lysozyme complex, eventually causing thinning of thick albumen and a decrease in HU values. Thus, albumen thinning is an intrinsic self-degrading property that causes changes in the content and structure of albumen during storage. For example, the ovomucin content is higher in dense, thick albumen than in less viscous albumen [10]. Alterations in the structure and composition of albumen proteins may also influence their functional properties.

Albumen protein content and structure tends to change during storage and such changes can reduce related functional properties. For instance, there was a decrease in ovomucin content after six weeks of storage, while no significant variation was observed for ovalbumin and lysozyme [72]. Similarly, Wang et al. [10] reported that increased storage time decreased ovomucin content in albumen, and a corresponding decrease in the viscoelasticity of albumen was observed. This is in agreement with the study of Shan et al. [36], which reported a decrease in rheological properties of albumen due to reduced ovomucin content. Ovalbumin structure (α- and β-sheets) is altered during storage, which reduces foaming and emulsifying properties [73]. Increased ApH during extended storage degrades ovomucin content, making it difficult to extract ovomucin [12]. These findings highlight that albumen proteins are sensitive to storage time and temperature. It is imperative to preserve the content and structure of these proteins to extend the shelf life of albumen without the loss of its functional properties.

The biological functions of albumen, such as antioxidant and antimicrobial activities, may be reduced because of alterations in albumen structure due to storage time and temperature effects. Free amino acids are some of the main contributors to the antioxidant activity of eggs; total antioxidant capacity (TAOC) tends to decline during storage [74]. Antioxidant capacity is more sensitive to storage temperature than storage time. Liang et al. [75] reported that the rate of decline of TAOC was slower at refrigerated temperatures than at ambient temperature. In another study, Nimalartane et al. [74] revealed that antioxidant capacity was very stable during six weeks of storage at refrigerated temperatures. This is likely because free amino acids, responsible for oxidative stability, are more stable at lower temperatures. This could explain why lipid and protein peroxidation products are often higher in eggs stored at higher temperatures than at lower temperatures, indicating that a low storage temperature does not act as a catalyst for lipid and protein peroxidation.

**Figure 1:**
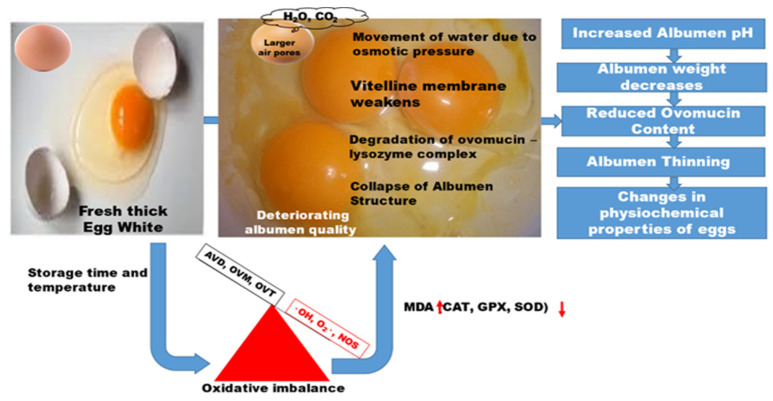
Changes in albumen structure during storage.

Structural changes in albumen occur due to storage time and temperature effects, culminating in a decline in albumen quality, which is characteristic of lost functional and technological properties (see Figure 1).

In conclusion, eggs are highly susceptible to deterioration during storage, owing to lipid peroxidation and protein denaturation. Strategies that would maintain albumen traits (AH, ApH, HU) in stored eggs similar to those of fresh eggs and help to inhibit biochemical reactions that lead to the collapse of albumen should be adopted. Albumen quality must be maintained during storage to extend its shelf life and increase the potential benefits to consumers and the food and health industries. The use of natural antioxidants that can be easily transferred to eggs and act as a natural shelf-life extender without residue effects is recommended. Investigating natural diets to preserve the internal quality of eggs may offer better chances of consumer acceptance and reduce storage costs.

## 3. Preservation of Albumen Shelf Life and Quality: Which Way?

Eggs contain a significant proportion of polyunsaturated fatty acids (PUFAs), mainly C18:2_n−6_, which make them highly susceptible to peroxidation during storage [2]. During storage, ammonia and hydrogen sulfide are synthesized by the enzymatic degradation of proteins and fats in the egg contents [38]. Hence, lipid and protein peroxidation occurs, which increases levels of oxidation products, including malondialdehyde (MDA), volatile basic nitrogen (VBN), and carbonyl content. Increased levels of these products reduce the total antioxidant capacity of the albumen and act as catalysts for biochemical reactions within the albumen, which adversely influence functional and sensory attributes.

In addition, animals may be exposed to oxidative stress owing to sickness, nutrition, or the environment. Oxidative stress leads to the release of hydroxyl and superoxide radicals that induce cellular degeneration, DNA damage, and apoptosis. Toxins from these abnormalities may be transferred to the eggs, thereby reducing their antioxidant capacity [76]. Various approaches, including storage environment modification or nutrition of laying hens, are targeted at enhancing albumen antioxidant capacity and retaining functional properties.

### 3.1. Storage Environment Modification

Egg preservation has been approached in various ways, with varying effects and limitations. Decontamination of eggshells is the first line of defense to avoid alteration of the internal components of the egg. Washing eggshells may reduce the microbial load on the surface but it can leave residues on the eggshell and damage the cuticle layer, facilitating microbial penetration into egg contents [11,77]. The use of pulsed light for egg preservation appears to be a useful decontamination method because the egg cuticle layer is protected and can be used to extend shelf life without residue issues. Moreover, no effect on the albumen quality was observed [18]. Approaches such as hot air pasteurization [78], ozone, and radiation treatments [19] have been used to decontaminate shells successfully. However, nutritional and functional properties of the egg may be altered. Pasteurization may be an acceptable approach, but it may reduce the functional quality of egg components [79], although Yang and Geveke [80] suggested that pasteurization with hot water spraying combined with radio frequency can be used for egg shell safety, while the textural property of the albumen was improved. Other interventions, including ultrasound treatment [4], modified atmosphere packaging (MAP) [20], slightly acidic electrolyzed water (SAEW) [21], electrolyzed water, and ultraviolet light [69], preserved albumen quality during storage. Most of these approaches may preserve albumen quality during storage but may be less feasible due to the skilled labor required to carry out the operations and may entail substantial financial costs. Natural products have been used as shell coatings to preserve albumen quality during storage. Coating with shellac [22] and glycerin oil [23] exerts an excellent sealing effect on eggshell pores, which reduces the escape of CO_2_, thereby controlling the internal environment.

These systems alone may not sufficiently suppress the generation of oxygen radicals. Exogenous antioxidants are essential for inhibiting or reducing oxidative damage in humans and animals. Plants or extracts can inhibit or slow down the rate of chemical deterioration of foods during storage because antioxidant compounds can be effectively transferred to the food system [81,82]. Natural antioxidants scavenge reactive oxygen species and augment the antioxidant potentials of food. The study of Varzaru et al. [83] reported higher vitamin E levels in the eggs of laying hens fed on tomato peel than in those of the control group. Eggs can be biofortified with plant bioactive compounds, which would enhance their nutritive value [84]. Herbs contain numerous glycosidically bound and non-volatile constituents that exert biological effects after enzymatic hydrolysis [85], which could explain their positive effects on animal performance and animal products. Therefore, it is necessary to explore natural dietary antioxidants that can reduce lipid and protein peroxidation to maintain the albumen quality of stored eggs and improve the nutritive value of eggs.

### 3.2. Translation of Diet Fed to Laying Hens into Improved Egg Quality during Storage

Supplementation of laying hen diets with antioxidants can remarkably affect egg quality [81]). Previously, common synthetic antioxidants, such as butylated hydroxyanisole (BHA), butylated hydroxytoluene (BHT), and tertiary butylhydroquinone (TBHQ), have been used to inhibit peroxidation and preserve product quality. However, consumer concerns over the residue effects and toxicity of these synthetic compounds have limited their use [86]. A herbal essential oil mixture was found to provide the highest antioxidant capacity in fresh eggs compared to synthetic BHT or the control [87]. Phytogenics, as natural antioxidants, can be used as safe feed additives in the diets of laying hens to maintain animal health and reduce adverse effects of storage by extending the shelf life of eggs. Most aromatic and medicinal plants contain secondary metabolites (flavonoids, flavanones, phenols, carotenoids, and saponins) and have been suggested to possess antioxidant and antimicrobial properties. These natural antioxidants may act via different pathways, including proenzyme inhibition, singlet oxygen activation, and transition metal chelation [88]. Vitamin E and polyphenols prevent the formation of lipid hydroperoxide by breaking the chain of lipid peroxidation in the cell membranes. Organic forms of zinc may also be advocated, since zinc, although not an antioxidant, prevents the formation of free reactive oxygen radicals owing to its antagonistic effect on the catalytic properties of redox-active transition metals (Fe and Cu) [89]. The effects of natural antioxidants supplemented in the diets of laying hens on albumen quality during storage are presented in Table 4.

The capacity of natural antioxidants to extend the shelf life of albumen and preserve its quality during storage is reflected in HU values, albumen pH, and height, these having been found to be similar to those of fresh eggs, with lower levels of oxidative products and enhanced total antioxidant capacity.

Albumen pH increased during storage, and a high storage temperature acted as a catalyst for this increase. Such increases have been found to alter the albumen bicarbonate buffer system, which consequently causes the deterioration of albumen quality during storage. Essential oil [94], natural astaxanthin [32], pumpkin seed meal [30], and strawberry leaf extract [45] lowered albumen pH compared to controls. The lower pH during storage may be linked to the capacity of these antioxidants to oxidize the hydroxyl amino acid components of the albumen. The low pH would inhibit biochemical reactions that would lead to collapse of the albumen structure [67]; hence, maintaining the pH level similar to that of fresh eggs aids in preserving albumen stability. Lowering the pH slows down the rate of albumen liquefaction, thereby maintaining HU values and albumen quality.

The Haugh unit value, an indicator of albumen quality, is influenced mainly by storage time and temperature. Reduced HU values during storage indicate a loss in albumen quality, and when HU values of stored eggs are similar to those of fresh eggs, this suggests that albumen quality is preserved, with extended shelf life. Previous studies [33,54,55,96] have demonstrated that supplementation with natural plant antioxidants in the diets of laying hens reduced the rate of decline in the HU values of stored eggs and preserved the shelf life of albumen. Natural plant products, such as natural astaxanthin [32], pumpkin seed meal at 9% [30], Chinese herbal extracts [93], marigold extract [49], and tea polyphenols [29], maintained HU values during storage compared to controls. In another study, pomegranate molasses enhanced HU values compared to controls, but not beyond ten days during 30 days of storage [55]. In addition, dietary peptides [98] and natural vitamin E antioxidants [99] slowed the rate of HU decline in stored eggs and preserved their shelf life. However, graded levels of green tea [100], fermented pine (*Pinus densiflora*) needle extract [60], ginseng leaf extract [47], strawberry guava extract [45], and Chinese herbal mix supplemented at 3% [101] did not influence the HU values of stored eggs. In contrast, the addition of grape pomace flour significantly decreased the HU value of stored eggs compared with that of the control [102]. Oils from plants used to supplement diets maintained the HU values of stored eggs compared to controls. The HU value for eggs from hens fed a crude palm oil (CPO) diet was higher when the storage period was extended compared to soybean oil [103], and, in the same vein, rapeseed oil [33] and oregano essential oil [94] enhanced HU values. Natural antioxidants in the form of organic trace elements chelated with amino acids and probiotics extend the shelf life of albumen during storage. For example, carbon chelates (CAPC) [58] improved the HU value of eggs stored at 25 °C for 21 d compared to controls, 100 mg/kg zinc–methionine [104] and selenium probiotics [105] improved the HU value in eggs stored for 15 days and 6 days, respectively. In another study, selenized glucose at 10 mg/kg enhanced HU values by 17% compared to controls [54]. In addition, linseed powder enhanced the albumen weight of eggs stored for 28 days at 4 °C compared to controls (34 g vs. 31 g [106]. When HU values are maintained during storage, the functional properties of albumen are consequently preserved. However, plants extracts (marigold and paprika) [92] and butyric acid from plants [57] had no influence on albumen quality during storage. The discrepancies observed with respect to the mitigation effects of these natural antioxidants could be due to the nature, inclusion level and level of antioxidant capacity.

Functional properties of albumen can be enhanced when albumen quality, reflected in HU values, is stable. Novel phytoadditives (bilberry leaves, walnut leaves, and sea buckhorn pomace) enhance albumen viscosity, thus preserving the rheological properties of eggs stored at 5 °C for 28 d [107]. Green tea in the diet of laying hens increases albumen viscosity in stored eggs [29]. The improved technological properties may be due to the capacity of antioxidants to enhance albumen height and HU value. There is limited information on the influence of natural antioxidants on functional properties of albumen during storage; this is as subject that may require further investigation.

During storage, the food system undergoes lipid and protein peroxidation, culminating in the release of peroxidation products that may adversely influence the functional and sensory attributes of food commodities. Natural antioxidants have been found to slow the peroxidation process in albumen and whole eggs during storage. Dietary tea polyphenols increased oxygen radical scavenging activity in the albumen of stored eggs [29,108]. Pumpkin seed meal at 9% increased the antioxidant capacity of the albumen in eggs stored at 5 °C and 21 °C for 28 d by reducing lipid peroxidation and protein denaturation [30]. Similarly, tomato peels and rosehip seeds [83], black tea waste [109], and marigold extract [49] supressed lipid peroxidation, as reflected in lower values of MDA, thereby enhancing the antioxidant capacity of eggs. In one study, 0.20 g/kg of yellow strawberry guava leaf extract reduced lipid peroxidation activity (149.53 vs. 401%) and increased TAOC (1.10 vs. 0.24%) in eggs stored for 28 d at room temperature [45]. Additionally, acai flour, a fruit byproduct rich in anthocyanins, reduced lipid peroxidation and enhanced the antioxidant capacity of stored eggs at 27 °C for 28 d [31]. Lee et al. [53] reported that lotus leaf extract reduced TBARS and VBN, whereas DPPH radical scavenging activity increased in the treated group compared to the control. The enhanced antioxidant capacity in stored eggs is due to the antioxidant action of the bioactive compound which prevented albumen degradation.

Results from the reviewed literature suggest that selenium (Se) can enhance the oxidative stability of eggs during storage. In one study, Zhao et al. [54] reported that eggs from animals fed selenized glucose and stored at 25 °C for 14 d had lower MDA values compared to controls, indicating that lipid peroxidation was suppressed to a certain degree.

Selenomethionine, an organic form of selenium, reduced lipid peroxidation via the enhanced activity of the antioxidant enzymes glutathione peroxidase “GSH-Px” and superperoxide dismutase “SOD” in eggs stored for 21 d at 25 °C [102]. Dietary organic selenium preserves albumen quality during storage owing to the transfer of a certain amount of dietary Se into the egg and improves egg GPX activity, protecting eggs from free radical oxidation [105]. Se is an essential component of selenoproteins, such as GSH-Px, which catalyses the reduction of hydrogen peroxide and organic hydroperoxides at the crystal and mitochondrial matrix levels [110]. Hence, Se acts as a potent antioxidant that protects cells and tissues from the oxidative damage caused by lipid and protein peroxidation.

It could be inferred that albumen and, consequently, egg quality are improved when there is an increase in the content and activities of antioxidant enzymes and antioxidant indicators, such as total antioxidant capacity (T-AOC) and oxygen radical absorbance capacity (ORAC), while reducing power and MDA are decreased in egg white [111,112]. However, the reduced antioxidant capacity of albumen hastens egg deterioration during storage.

The mitigating effects of these natural antioxidants on albumen quality during storage hinge on the capacity of these plant-derived bioactive compounds to terminate free radical reactions and scavenge reactive oxygen species [113], improving the antioxidant capacity of albumen by increasing the activity of antioxidant enzymes. In one study, Florou-Paneri et al. [114] suggested that there is a transfer of antioxidants compounds from plants or extracts to eggs, protecting eggs from free radical oxidation and extending the shelf life of albumen. The natural antioxidants examined in this study may exert similar effects during storage but may have unique features that make them natural shelf-life extenders.

Phenols are excellent antioxidants and shelf-life extenders because large numbers of phenolic compounds in plant additives can be introduced into eggs and preserve albumen. Phenols tend to have the strongest antioxidant effects, being a group of chemical components consisting of one or more hydroxyl residues [115]. The different hydroxyl groups have aromatic and conjugated structures that are effective electron donors for scavenging free radicals [108]. The enhanced activity of polyphenols could occur at low concentrations in tissues; they appear to be sufficient to exert biological effects and are considered potent antioxidants [116].

Essential oils containing bioactive compounds, such as thymol, carvacrol, and rosmarinic acid, reduce lipid peroxidation. The antioxidant effect of oil is attributable to its capacity to delocalize unpaired electrons within the aromatic structures of phenolic substances, supplying hydrogen atoms or electrons to free radicals, which destabilizes the formation of hydrogen peroxides and scavenging chain-carrying peroxyl radicals [94]. There is a scarcity of information on the effect of different oils on the preservation of albumen during storage, which requires further investigation.

The capacity of organic trace elements and minerals to exert antioxidant effects and extend the shelf life of eggs could be linked to various factors; for instance, the ability of these mineral molecules to bind to organic molecules aids in preserving internal egg quality during the storage period, both at room and refrigerated temperatures; the capacity of dietary minerals to diminish the dissociation of ovomucin–lysozyme, a compound that maintains the integrity of albumen viscosity [117], and the high biological value of these trace elements; and zinc and selenium in their organic forms are easily transferred to the egg compared to inorganic sources [105]. These findings suggest that trace elements and minerals can reduce rates of albumen degradation and enhance albumen quality, thereby extending the shelf life of eggs during storage. Finally, nutritional interventions preserve albumen quality during storage, providing albumen of good quality, with benefits for the consumer and the food and health industries.

## 4. Conclusions and Future Perspectives

Albumen quality of stored eggs must be preserved because of its biological and technological properties. Albumen quality declines during storage due to reduced albumen protein content and protein–protein interactions. In past decades, research has focused on the performance and health of chickens, with less focus on how to maintain albumen quality in stored eggs using natural plant antioxidants. Various types of plant products have been demonstrated to influence albumen quality and provide a new nutritional strategy to preserve this animal product which is highly susceptible to deterioration over storage time. Biofortification of eggs with natural antioxidants in plants, animals, and organic elements tends to preserve eggs without danger of residue effects. Thus, going forward:

- Nutritional interventions with natural antioxidants from plants sources would help provide a pragmatic solution to declining albumen quality during storage, using natural products that raise no negative health concerns for consumers;
- The investigation of individual albumen protein changes during storage may provide a better understanding of the mechanisms involved;
- Investigation of new bioactive ingredients for the stabilization of liquid eggs may be exciting alternatives for maintaining oxidative stability and reducing heat treatment.

## Figures and Tables

**Table 1:** The effects of storage time and temperature on albumen pH.

Fresh	Stored	Temperature	Duration (Days)	References
7.92	9.29	22	63	[17]
8.07	8.88	5	63	[17]
8.46	9.31	22	28	[43]
8.48	9.07	5	28	[43]
8.66	9.01	4	28	[44]
8.56	9.21	25	28	[45]
8.4	9.26	5	28	[46]
8.6	9.41	22	28	[46]
8.53	9.4	4	28	[41]
8.22	9.63	20	28	[41]
8.05	9.48	20	42	[37]
7.54	8.54	23	28	[14]
7.54	8.26	4	28	[14]
8.8	9.12	10	28	[47]
8.66	9.43	21	30	[42]
7.5	9.56	24	35	[3]
8.03	8.82	25	30	[48]
8.03	6.8	12	30	[48]
8.2	9.4	30	9	[40]
8.68	9.25	4	28	[49]

**Table 2:** Effects of storage time and temperature on albumen height.

Fresh	Stored	Temperature	Duration (Days)	References
9.43	2	22	63	[17]
8.75	5.06	5	63	[17]
6.16	2.46	22	28	[43]
8.13	4.72	5	28	[43]
7.55	5.04	25	30	[48]
5.3	3.91	5	28	[46]
5.05	2.3	22	28	[46]
5.7	5.56	4	28	[14]
5.7	2.28	23	28	[14]
8.33	4.47	14	28	[23]
7.51	6.26	12	30	[48]
7.51	3.42	25	30	[48]
6.86	2.58	30	9	[40]
6.13	4.36	30	15	[53]
6.71	6.54	4	28	[35]
9.2	0	25	28	[35]
7.17	4.96	4	84	[38]
6.87	5.88	4	28	[49]
6.76	4.25	25	14	[54]
6.46	4.25	25	14	[55]

**Table 3:** (**a**) Effects of storage time and temperature (ambient) on Haugh unit values. (**b**) Effects of storage time and temperature (low) on Haugh unit values.

**(a)**					
**Fresh**	**Stored**	**Grade**	**Temperature**	**Duration (Days)**	**References**
81.99	58.46	B	20	42	[37]
81.23	58.93	B	24	35	[3]
85.04	50.08	B	25	30	[48]
81.39	37.55	C	30	9	[40]
79.21	62.82	A	30	15	[53]
80.17	50.04	B	24	42	[22]
95.69	0	na	25	56	[25]
89.94	0	na	25	28	[35]
98.6	39.36	C	22	63	[17]
75.93	38	C	22	28	[43]
85.04	65.56	A	25	30	[48]
82.17	23.94	C	21	28	[42]
83.6	0	na	25	56	[32]
67.04	32.71	C	22	28	[46]
94.7	77.57	AA	20	28	[41]
72.7	32.66	C	23	28	[14]
80.53	59.37	B	25	14	[54]
**(b)**					
**Fresh**	**Stored**	**Grade**	**Temperature**	**Duration (Days)**	**References**
80.95	79.48	AA	4	28	[35]
83.65	67.52	A	4	84	[38]
95.75	74.22	AA	5	63	[17]
75.47	64.47	A	5	28	[43]
76.66	56.1	B	4	30	[59]
82.04	75.85	AA	4	28	[48]
83.42	75.22	AA	4	56	[32]
70.86	56.41	B	5	28	[46]
93.58	87.57	AA	4	28	[41]
72.27	71.6	A	4	28	[14]
90.5	63.68	B	14	28	[23]
93.8	84.45	AA	10	28	[47]
85.04	77.17	AA	12	20	[48]
87.1	60.9	A	18	28	[60]

na, not available.

**Table 4:** Effects of natural antioxidants from plants on albumen quality during storage.

			Haugh Unit Values					
Storage Time (Days)	Storage Temperature	Diet and Feeding Time	Control	Treated	Grade	Bioactive Compounds	Main Effect	References
14	RT	100 mg/kg magnolol: 12 weeks	36.9	46.29	B	Source: magnolia plant bark: phenols	Extended shelf life	[90]
14	RT	Rapeseed oil: 12 weeks	70.22	78.11	AA	Erucic acid	Extended shelf life	[33]
15	RT	200 mg/kg NHDC neohesperidin dihydrochalcone): 10 weeks	46.42	66.65	A	Natural flavonoids	Extended shelf life	[91]
30	5	40 g/kg terebinth seed meal: 8 weeks	56.1	68.73	A	Tannins	Extended shelf life	[59]
14	28	0.8% of marigold and paprika extracts: 9 weeks	74.93	72.64	AA	Lutein and capsanthin	No influence on albumen quality	[92]
7	20	0.2% *Lonicera confusa* and Astragali Radix extracts: 12 weeks	55.24	59.19	B	Ellagic acid and chlorogenic acid	Extended shelf life	[93]
25	21	CAPC–organic zinc 43.7 mg/kg: 24 weeks	53.76	60.55	A	Zinc	Extended shelf life	[58]
25	21	50 mg oregano essential oil: 4 weeks	23.26	46.94	B	Thymol, carvacrol	Extended shelf life	[94]
56	4	*Mentha × piperita* extract at 100 mg/kg: 8 weeks	79.91	78.48	AA	phenols	Not significantly different from control	[82]
30	Natural state	800 mg resveratrol/kg. 8 weeks	50	70	AA	Phenols from grapes	Extended shelf life	[95]
28	21	9% pumpkin seed meal. 8 weeks	32.12	44.40	B	Lutein, α and β-carotene	Preserved albumen quality	[30]
28	5	9% pumpkin seed meal. 8 weeks	54.62	58.52	B	Lutein, α and β-carotene	Preserved albumen quality	[30]
56	25	160 mg natural astaxanthin/kg. 4 weeks	56.92	62.3	A	Lutein	Extended shelf life	[32]
56	4	160 mg natural astaxanthin/kg. 4 weeks	78.11	81.01	AA	Lutein	Extended shelf life	[32]
28	4	2 g marigold extract/kg diet. 4 weeks	75.85	76.38	AA	Lutein	Maintained stability	[49]
14	25	Vitamin E at 200 mg/kg. 8 weeks	40.63	50.21	B	Vitamin E	Extended shelf life	[96]
21	27	50 mg/kg curcumin: 8 weeks	31.8	42.76	B	Curcuminoids	Extended shelf life	[97]
14	25	Selenised glucose 10 mg/kg feed. 5 weeks	59.37	67.14	A	Selenium	Extended shelf life	[54]
10	4	0.50% of pomegranate molasses/drinking water: 4 weeks	83.17	88.95	AA	Phenols	Preserved albumen quality not beyond 10 days	[55]
28	18	2.5 mL, 5.0 mL of fermented pine needle extract (*Pinus densiflora*). 6 weeks	60.9	60.05	A	Polyphenols, essential oils	No influence on albumen quality	[60]
